# Discovery of Three New Phytotoxins from the Fungus *Aspergillus nidulans* by Pathway Inactivation

**DOI:** 10.3390/molecules24030515

**Published:** 2019-01-31

**Authors:** Lijuan Liao, Xiaolei Zhang, Yi Lou, Chengzeng Zhou, Qianqian Yuan, Jiangtao Gao

**Affiliations:** College of Life Sciences, Fujian Agriculture and Forestry University, Fuzhou 350002, China; liao_0909@fafu.edu.cn (L.L.); 2170517007@fafu.edu.cn (X.Z.); louyi032@fafu.edu.cn (Y.L.); 1170516008@m.fafu.edu.cn (C.Z.); 3170515037@m.fafu.edu.cn (Q.Y.)

**Keywords:** *Aspergillus nidulans*, pathway inactivation, phytotoxins, phytotoxic assay

## Abstract

Fungi are a source of novel phytotoxic compounds to be explored in the search for effective and environmentally safe herbicides. The genetic inactivation of the biosynthetic pathway of the new phytotoxin cichorine has led to the isolation of three novel phytotoxins from the fungus *Aspergillus nidulans*: 8-methoxycichorine (**4**), 8-*epi*-methoxycichorine (**5**), and *N*-(4’-carboxybutyl) cichorine (**6**). The structure of the new compounds was clearly determined by a combination of nuclear magnetic resonance (NMR) analysis and high-resolution electrospray ionization (HRESIMS). The phytotoxic bioassay was studied on leaves from *Zea mays* and *Medicago polymorpha* L. at the concentration of 5 × 10^−3^ M by using a moist chamber technique. Novel phytotoxins 8-methoxycichorine (**4**), 8-*epi*-methoxycichorine (**5**), and *N*-(4’-carboxybutyl) cichorine (**6**) exhibited a better phytotoxic effect than cichorine.

## 1. Introduction

Herbicides have been used widely in the field of agriculture to kill weed pests and increase crop yield production. However, most of the available herbicides are synthetic chemical compounds, causing serious pollution of the ecological system due to their large-scale usage and difficult degradation [1,2]. Meanwhile, herbicide resistance is increasing in weed control after a long history of the application of existing commercial herbicides [3,4,5]. Therefore, the exploration of new herbicidal agents is of increasing importance. 

To date, more than 400 phytotoxins have been isolated and structures identified from microbial sources, which possess different chemical characterizations [6,7]. With great structural diversity and novel mechanisms compared to synthetic herbicides, naturally occurring compounds from microbes have been ideal candidates for new herbicides. They have been used as natural herbicides directly, as leading compounds, or for simultaneous applications with a low dose of synthetic herbicides to achieve a better treatment outcome [8]. Several compounds have been the product of successful commercial herbicides, or the prototype of new herbicides against several kinds of weeds worldwide. Glufosinate, also known as phosphinothricin, is produced by several species of *Streptomyces* bacteria. It is one of the most successful commercial herbicides because it is a broad-spectrum herbicide, and is widely used to control important weeds such as *Ipomoea aquatica*, *Sesbania aculeata* (Wild.) Pers., *Polygonum pensylvanicum* and *Cyperus esculentus* [9,10,11,12]. Hydantocidin, a spironucleoside from *Streptomyces hygroscopicus*, and its synthetic analogues have been patented as herbicides [13,14].

Cichorine is a novel phytotoxin active against knapweed, corn, and soybeans. It was discovered from several fungi including *A. nidulans* and *Alternaria cichorii*, which produces foliar blight in the important pest Russian knapweed. We formulated the hypothesis that cichorine is an important lead compound for new herbicide development because its compact, functionalized isoindolin-1-one framework is extremely attractive in the field of drug discovery [15]. Therefore, it is highly important to discover structural analogues of cichorine for new herbicides. In our previous research, a cluster of seven genes including non-reducing polyketide synthase AN6448 was confirmed as a requirement for the biosynthesis of cichorine in the strain *A. nidulans*. Interestingly, cichorine produced by this pathway could be the precursor to other interesting compounds, as the deletion of AN6448 led to no production of the NRPS peptide aspercryptin. This provides us with a powerful platform to discover new analogues of cichorine with good herbicide activity. The occurrence of many tailoring enzymes in the strain *A. nidulans* makes us believe that some cichorine-derived compounds could be biologically synthesized in this fungus [16]. In order to isolate and identify the cichorine derivatives, we selected the engineered *A. nidulans* LO8030, in which the gene clusters responsible for the biosynthesis of most of the known secondary metabolites in the wild type were deleted to reduce metabolite background in *A. nidulans* and minimize the rediscovery of known compounds. The gene deletion of the PKS gene AN6448∆ (Δ*pkbA*) from LO8030 was conducted because the PKS gene is responsible for the first step in the biosynthesis of cichorine, and therefore it is crucial for the biosynthesis of any cichorine-derived compound. High-performance liquid chromatography coupled with a UV detector (HPLC-UV) profile analysis of the ethyl acetate (EtOAc) extract from the parental and Δ*pkbA* strains resulted in the discovery of six compounds, including 3,6-dimethyl-4-hydroxy-2-methoxybenzaldehyde (**1**) [17], nidulol (**2**) [18], cichorine (**3**) [15], 8-methoxycichorine (**4**), 8-*epi*-methoxycichorine (**5**), and *N*-(4’-carboxybutyl) cichorine (**6**). Among the metabolites, compounds (**4**), (**5**), and (**6**) were determined as three new cichorine analogues with potent phytotoxicity. Here, we report the isolation, purification, structure determination, and biological assay of the isolate compounds by pathway inactivation. 

## 2. Results and Discussion

**Figure 1:**
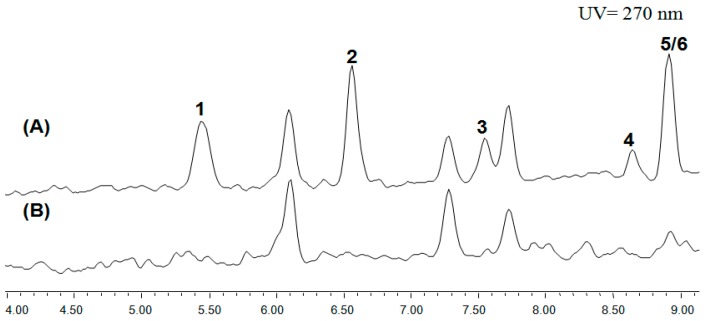
The comparison of HPLC-UV analysis of secondary metabolites from the parental strain (**A**) and the Δ*pkbA* deletion strain (**B**) of *Aspergillus nidulans* LO8030.

**Figure 2:**
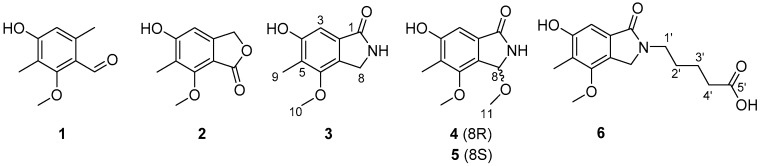
The structures of compounds **1**–**6** isolated from *A. nidulans* LO8030.

The AN6448 deletion mutant was generated by the homologous transformation of the *A. nidulans* strain LO8030. The AN6448 gene was replaced by a pyrG selectable marker from *A. fumigatus* 293, as seen in Figure S1A. The resulting transformants were confirmed by diagnostic PCR, as shown in Figure S1B. The HPLC results showed that the cichorine was drastically eliminated in the positive deletion mutant in comparison with the wild-type LO8030, which was designated as the control in Figure 1. This was consistent with the previous study, which confirmed the involvement of the AN6448 gene in cichorine biosynthesis. Consequently, we discovered six compounds (**1**–**6**), three of which are new ones (**4**–**6**) in the ΔAN6448 mutant, as shown in Figure 2.

**Figure 3:**
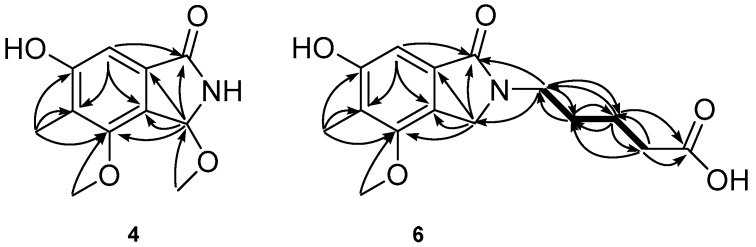
The COSY (bold line) and selected gHMBC (arrows) correlations of compounds **4** and **6**.

The molecular formula of 8-methoxycichorine (**4**) was deduced to be C_11_H_13_NO_4_ by high-resolution electrospray ionization (HRESIMS) analysis. The six degrees of unsaturation indicated the presence of one aromatic ring. The UV spectrum showed the absorption at 215, 259, and 298 nm, which is in accordance with the appearance of the aromatic ring in compound **4**. The ^13^C-NMR data of this compound showed a carbonyl carbon signal at the δ_C_ value of 172.5 and a benzene ring with carbon signals at δ_C_ values of 159.8, 156.5, 132.7, 123.6, 122.3, and 104.6, as shown in Table 1. In addition to the downfield carbon signals, the ^1^H-NMR and ^13^C-NMR data also showed an *N*-substituted aliphatic methine (δ_H_ 6.04, δ_C_ 85.3), a methyl group (δ_H_ 2.13, δ_C_ 9.53), and two methoxy groups (δ_H_ 3.18, δ_C_ 52.2 and δ_H_ 3.96, δ_C_ 60.1, respectively). By a series of ^1^H detected heteronuclear multiple bond for correlations (HMBC) between H-3 to C-1/C-5/C-6/C-7, H-8 to C-1/C-2/C-6/C-7, 5-CH_3_ protons to C-4/C-5/C-6, 6-OCH_3_ protons to C-6, and 8-OCH_3_ protons to C-8 (Figure 3), the planar structure of compound **4** was determined as 8-methoxycichorine, which was the derivative of cichorine (**3**). The configuration of the asymmetric carbon center at C-8 is discussed below.

The molecular formula of 8-*epi*-methoxycichorine (**5**) was deduced to be C_11_H_13_NO_4_ by HRESIMS analysis, which was identical to **4**. The planar structure of this compound was determined to be the same as **4** based on the ^1^H-NMR and ^13^C-NMR results (Table 1). Although the comparison of ^1^H-NMR and ^13^C-NMR chemical shifts between compounds **4** and **5** had no obvious difference, the optical rotations of both compounds were the opposite (the values for **4** and **5** were +5.3 and −4.1, respectively), suggesting these compounds exhibited an epimeric relationship at C-8.

**Figure 4:**
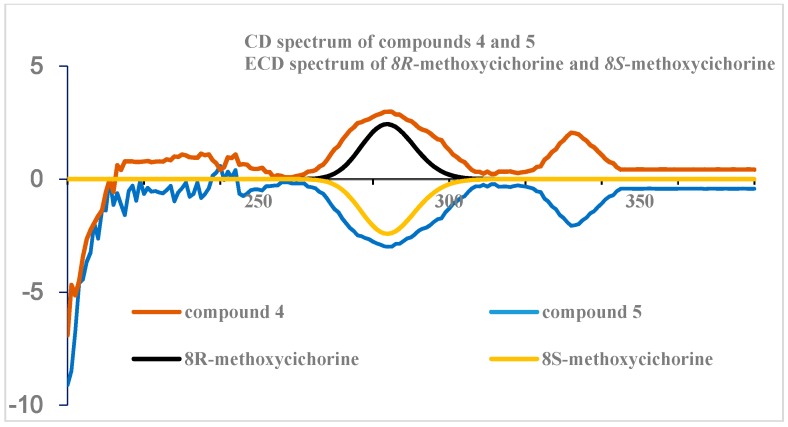
The comparison of CD and ECD spectra.

The absolute configuration at C-8 was eventually defined by circular dichroism (CD) measurements and ECD calculations. The CD profiles of **4** and **5** had opposite profiles at approximately 284 nm that were highly consistent with the ECD calculation for the model of 8*R* and 8*S*, respectively, as shown in Figure 4. Additionally, the ECD spectrum for compound **4** is in accordance with the 3-methoxyporriolide [19].

The molecular formula of *N*-(4’-carboxybutyl) cichorine (**6**) was deduced to be C_15_H_19_NO_5_ by the HRESIMS analysis. The NMR data of this compound was very similar to that of cichorine (**3**), with the most noticeable difference being the replacement of the carboxybutyl group at NH, which had signals at δ_C_ values of 43.2, 35.3, 28.9, 23.6, and 177.3, and δ_H_ values of 3.61, 2.33, 1.73, and 1.62. This interpretation was readily confirmed by a combination of 2D NMR experiments, in which all of the proton–proton and carbon–proton correlations obtained and supported the placement of a carboxybutyl group at the N position (Figure 3). The carboxylic signal 177.3 in ^13^C-NMR was assigned to C-5’ based on the HMBC correlations between H-3’ (δ_H_ 1.62) to C-5’ (δ_C_ 177.3) and H-4’ (δ_H_ 2.33) to C-5’ (δ_C_ 177.3). Thus, *N*-(4’-carboxybutyl)cichorine (**6**) was structurally elucidated to be an *N*-carboxybutyl derivative of **3**. 

The plausible biogenetic pathway for cichorine (**3**) proposed by the Kawai Ken-ichi group is as follows [18]. The first step is the formation of 3-methylorsellinate through the acetate-malonate pathway, followed by cyclization and the introduction of the C_1_ unit at the C-5 position, followed by *O*-methylation at C-6. The oxidation of the methyl group at C-1 of 3-methylorsellinate and cyclization makes compound **3**. Compound **3** is the key intermediate, and transforms into **4**, **5**, and **6** by *O*-methylation at C-8 and the introduction of a carboxybutyl group at the N position in this study, respectively.

The phytotoxic activities of zinniol-related compounds have been reported, and the relationship between their structure and phytotoxicity have been studied. However, the relationship between the structure and phytotoxicity of cichorine-derived compounds with an isoindolinone skeleton has rarely been discussed. Previous studies showed that the alkylation of the 6-OH group of cichorine (**3**) abolished the phytotoxicity in a leaf-spot assay [20]. We therefore investigated the activity of isolated compounds by a leaf-spot assay. Compounds **1** and **2** were inactive. The activities of **3**–**6** are presented in Table 2. Compounds **4** and **5**, which have the methoxy group at C-8, exhibited more potential activity than cichorine (**3**), indicating that structural modification of position C-8 in cichorine (**3**) is important for improving the phytotoxicity of this type of compound. Compounds **4** and **5** exhibited similar activities toward the tested plant. Interestingly, compound **6** showed stronger phytotoxicity than cichorine (**3**), as seen in Table 2, suggesting that the *N*-alkyl and carboxylic acid groups contributed to the phytotoxicity. However, it should be kept in mind that the relationship between the structure and phytotoxicity of cichorine-derived compounds depends on the species and organisms of the plants. For example, in a seedling-growth assay against stone leek and lettuce, *N*-alkyl and hydroxyl groups enhanced the activity, but sulfonation almost abolished the activity when the 6-OH group of cichorine (**3**) was alkylated. These results are important for the further modification of cichorine to discover the potential phytotoxins as herbicide candidates. 

## 3. Materials and Methods 

### 3.1. General Experimental Procedures

Optical rotations were measured on a JASCO P-1020 polarimeter (JASCO, Tokyo, Japan) using a 1 cm cell. UV spectra were acquired with a Hitachi U-3010 spectrophotometer (Hitachi, Tokyo, Japan). NMR spectra were recorded on a JEOL 400 spectrometer (JEOL, Tokyo, Japan). Proton and carbon NMR spectra were measured in MeOH-*d*_4_ and CDCl_3_ solutions at 400 and 100 MHz, respectively. High-resolution electrospray ionization mass spectrometry (HRESIMS) data were acquired using the Agilent 1290 Infinity LC System (Agilent, Palo Alto, USA) coupled with the Agilent 6540 Ultra High Definition Accurate-Mass Q-TOF LC/MS (Agilent, Palo Alto, USA). HPLC measurements were performed on an Essentia Pre LC-16P (Essentia, Kyoto, Japan) equipped with a UV detector. All solvents used were of spectroscopic grade or distilled from glass prior to use. 

### 3.2. Molecular Genetic Methods

The gene deletion was performed using established gene targeting procedures [21]. The 1267 bp upstream fragment and 928 bp downstream of the PKS *pkbA* (AN6448) involved in cichorine biosynthesis were amplified from *A. nidulans* LO8030 genomic DNA using PCR. Primers used in this study are listed in Table S1. The pyrG gene was amplified, as a selectable marker cassette from *A. fumigatus* 293 genomic DNA. The two amplified flanking sequences and the pyrG gene were fused together by PCR using nested primers. Production of protoplasts and transformations were carried out as previously described. The strain LO8030 carrying four mutations (i.e., pyroA4, riboB2, pyrG89, and nkuA::argB) was used as the recipient strain. For selection of transformants, 0.5 µg/mL pyridoxine and 2.5 µg/mL riboflavin were added to selection plates. For further verification, diagnostic PCR was performed two more times, as described using the primers located inside the targeted gene [16], as shown in Table S1.

### 3.3. Fermentation and Isolation 

The *A. nidulans* strain LO8030 and Δ*pkbA* were cultured on YAG agar plates (5 g yeast extract, 20.0 g d-glucose, 15.0 g agar in 1 L distilled water, and 1 mL Hutner’s trace element solution) which were supplemented with riboflavin (2.5 mg/L), pyridoxin (0.5 mg/L), uracil (1 g/L), and uridine (10 mM). The fermentation was conducted at 37 °C for 7 days. Then, the plates were extracted with MeOH three times. The solvent was evaporated to obtain an organic extract. The extract was separated by C_18_ reversed-phase vacuum flash chromatography using a sequential mixture of MeOH and H_2_O as eluents (eight fractions in gradient, H_2_O‒MeOH, from 100:0 to 0:100), acetone, and finally EtOAc. On the basis of the results of the ^1^H-NMR analysis, the fraction eluted with H_2_O‒MeOH (60:40) and H_2_O‒MeOH (40:60) were chosen for separation. The fraction eluted with H_2_O‒MeOH (60:40) (3.32 g) was separated by semipreparative reversed-phase HPLC (from 70:30 to 40:60 in 40 min with H_2_O‒MeOH as the solvent system, 2.0 mL/min) to afford compounds **1** (2.0 mg), **2** (2.6 mg), and **3** (5.9 mg), respectively. The fraction eluted with H_2_O‒MeOH (40:60) (0.46 g) was separated by LH-20 (H_2_O‒MeOH, 40:60, 3 drops/min) followed with purification by reversed-phase HPLC analysis with H_2_O-MeCN as eluent to afford a mixture of **4** and **5** (3.6 mg), and compound **6** (0.9 mg). Compounds **4** (1.1 mg) and **5** (0.9 mg) were separated by a chiral column (Phenomenex lux cellulose-3, H_2_O–ACN as solvent system, from 90:10–0:100 in 30 min, 1.0 mL/min). 

**3,6-dimethyl-4-hydroxy-2-methoxybenzaldehyde** (**1**): pale yellow amorphous solid; UV (EtOH) *λ*_max_ (log ε): 232 (4.10), 284 (4.15) nm; ^1^H-NMR (400 MHz, CDCl_3_) *δ*: 10.35 (1H, s, 1-CHO), 6.97 (1H, br s, 4-OH), 6.52 (1H, s, 5-H), 3.83 (3H, s, 2-OMe), 2.54 (3H, s, 6-Me), 2.18 (3H, s, 3-Me). Compound **1** was identified by comparison of the ^1^H-NMR with the authentic sample.

**nidulol** (**2**): pale yellow amorphous solid; UV (EtOH) *λ*_max_ (log ε): 215 (4.55), 256 (3.80), 303 (3.47) nm; HRESIMS, *m*/*z* 195.0654 [M + H]^+^ (Calcd. for C_10_H_11_O_4_, 195.0657). Compound **2** was identified by comparison of the ^1^H-, ^13^C-NMR chemical shifts and HRESIMS with the authentic sample.

**cichorine** (**3**): pale yellow amorphous solid; UV (MeOH) *λ*_max_ (log ε): 212 (4.00), 252 (3.32), 295 (3.01) nm; HRESIMS, *m*/*z* 194.0815 [M + H]^+^ (Calcd. for C_10_H_12_NO_3_, 194.0817). Compound **3** was identified by comparison of the ^1^H-, ^13^C-NMR chemical shifts and HRESIMS with the authentic sample.

**8-methoxycichorine** (**4**): yellow amorphous solid; m.p. 287 °C; ${\left[\alpha \right]}_{D}^{25}$ + 5.3 (*c* 0.5, MeOH); UV (MeOH) *λ*_max_ (log ε) 215 (4.07), 259 (3.37), 298 (3.10) nm; CD (MeOH) λ (∆ε) 284 (+3.05), 352 (+2.06) nm; IR (ZnSe) ν_max_ 3394 (br), 1665, 1572 cm^−1^; ^1^H- and ^13^C-NMR data, see Table 1; HRESIMS, *m*/*z* 246.0751 [M + Na]^+^ (Calcd. for C_11_H_13_NO_4_Na, 246.0742).

**8-*epi*-methoxycichorine** (**5**): yellow amorphous solid; m.p. 287 °C; ${\left[\alpha \right]}_{D}^{25}$ − 4.1 (*c* 0.5, MeOH); UV (MeOH) *λ*_max_ (log ε) 215 (4.07), 259 (3.37), 298 (3.10) nm; CD (MeOH) λ (∆ε) 284 (−3.02), 352 (−2.08) nm; IR (ZnSe) ν_max_ 3393 (br), 1666, 1579 cm^−1^; ^1^H- and ^13^C-NMR data, see Table 1; HRESIMS, *m*/*z* 246.0751 [M + Na]^+^ (Calcd. for C_11_H_13_NO_4_Na, 246.0742).

***N*-(4’-carboxybutyl)cichorine** (**6**): white amorphous solid; m.p. 470 °C; UV (MeOH) *λ*_max_ (log ε) 213 (4.03), 254 (3.33), 295 (3.05) nm; IR (ZnSe) ν_max_ 3374, 1778, 1662, 1572 cm^−1^; ^1^H- and ^13^C-NMR data, see Table 1; HRESIMS, *m*/*z* 294.1348 [M + H]^+^ (Calcd. for C_15_H_20_ NO_5_, 294.1341).

### 3.4. Computational Analysis 

The ground-state geometries were optimized with density functional theory (DFT) calculations using Turbomole 6.5 with the basis set def-SV(P) for all atoms at the DFT level, using the B3LYP functional. The ground states were further confirmed by a harmonic frequency calculation. The calculated ECD data corresponding to the optimized structures were obtained using time-dependent density-functional theory (TDDFT) with the basis set def2-TZVPP for all atoms at the DFT level, using the B3LYP functional. The ECD spectra were simulated by overlapping each traction, where *σ* is the width of the band at 1/e height. t*Ei* and *Ri* are the excitation energies and rotatory strengths for transition *i*, respectively. In the current work, the value of σ was fixed at 0.09 eV.

$$∆\in \left(E\right)=\frac{1}{2.297\times 10^{-29}}\frac{1}{\sqrt{2\pi \sigma }}\sum_{i}^{A}∆E_{i}R_{i}e^{\left[-\frac{{\left(E-∆E_{i}\right)}^{2}}{{\left(2\sigma \right)}^{2}}\right]}$$

### 3.5. Phytotoxic Bioassay

The phytotoxic activities of the cichorine analogues were tested on the cut leaves using a moist chamber technique as described in reference [15]. The leaves were from *Zea mays* and *Medicago polymorpha* L., respectively. All the used leaves were cut from three or four-week-old plants with the size 1.5 cm × 1.5 cm. All the tested compounds were prepared with the concentration at 5 × 10^−3^ M. The pricks were made using the tip of a 10 µL Hamilton syringe, and a droplet (10 µL) of test solution was applied in the center of the leaves from the upper side. The diameters of the lesions were measured after 48 h of incubation in a moist, sterile chamber at 25 °C. The compounds were prepared in 2% EtOH. The cichorine was chosen as the positive control and 2% EtOH was tested as the negative control.

### 3.6. Statistical Analysis

Experiments were carried out at least in triplicate, and results were expressed as the mean ± standard deviation (SD). Statistical analysis was performed using the SPSS statistical package for the Social Sciences software. A *p* value of < 0.05 was considered to be statistically significant.

## 4. Conclusions

In summary, we reported the isolation, purification, structural elucidation, and bioassay of six cichorine-derived metabolites from the fungus *A. nidulans* LO8030 by using pathway inactivation. Three compounds were identified as new natural products with an isoindolinone skeleton and exhibited better phytotoxicity than cichorine on plant leaves, indicating that cichorine derivatives would be promising in the course of developing novel herbicides.

## Figures and Tables

**Table 1:** Nuclear magnetic resonance (NMR) data of compounds **4**, **5**, and **6** in methanol-*d*_4_.

Position		4			5			6	
	δ_C_	δ_H_ (*J* in Hz)	HMBC	δ_C_	δ_H_ (*J* in Hz)	HMBC	δ_C_	δ_H_ (*J* in Hz)	HMBC
1	172.5	-		172.5	-		170.8	-	
2	132.7	-		132.8	-		132.7	-	
3	104.6	6.87 (1H, s)	1, 2, 5, 6, 7	104.6	6.87 (1H, s)	1, 2, 5, 6, 7	104.5	6.89 (1H, s)	1, 2, 5, 7
4	159.8			159.9			158.4		
5	122.9			122.9			122.3		
6	156.5			156.5			155.2		
7	123.6			123.6			123.4		
8	85.3	6.04 (1H, s)	1, 2, 6, 7	85.3	6.05 (1H, s)	1, 2, 6, 7	49.7	4.54 (2H, s)	1, 2, 6, 7
9	9.5	2.13 (3H, s)	4, 5, 6	9.5	2.14 (3H, s)	4, 5, 6	9.5	2.14 (3H, s)	4, 5, 6
10	60.1	3.96 (3H, s)	6	60.1	3.96 (3H, s)	6	60.0	3.90 (3H, s)	6
11	52.2	3.18 (3H, s)	8	52.2	3.18 (3H, s)	8			
1’							43.2	3.61 (2H, t, *J* = 7.0 Hz)	1, 8, 2’, 3’
2’							28.9	1.73 (2H, m)	1’, 3’
3’							23.6	1.62 (2H, m)	1’, 2’, 4’, 5’
4’							35.3	2.33 (2H, t, *J* = 7.1 Hz)	2’, 3’, 5’
5’							177.3		

**Table 2:** Leaf-spot bioassays (mm necrotic lesion) of compounds **3**–**6** from the fungus *A. nidulans* tested at 5 × 10^−3^ M.

Phytotoxins	*Zea mays*	*Medicago polymorpha* L.
Cichorine (**3**)	8.2 ± 0.5 *^a^*^,^*	7.5 ± 0.7
**4**	9.0 ± 0.8	9.3 ± 0.3
**5**	8.9 ± 0.6	9.4 ± 0.5
**6**	9.5 ± 0.5	8.8 ± 0.2

*^a^* Each value is shown as the average ± standard deviation. (*) *p* < 0.05.

## Supplementary Materials

The Supplementary Materials are available online. Table S1 PCR primers used in this study. Figure S1 Diagnostic PCR strategy and results. Figures S2–S8 The NMR, UV spectra and HREISMS ion mode for compound **4**. Figures S9–S13 The NMR spectra for compound **5**. Figures S14–S20 The NMR, UV spectra and HREISMS ion mode for compound **6**.
