# Supplementary material for: Discovery of Three New Phytotoxins from the Fungus Aspergillus nidulans by Pathway Inactivation
Source: Molecules. 2019 Jan 31;24(3):515. doi: 10.3390/molecules24030515 (PMC6384659; doi:10.3390/molecules24030515)
Supplement: Supplementary file 1 [file molecules-24-00515-s001.pdf]

Supporting Information

Discovery of three new phytotoxins from the fungus *Aspergillus nidulans* by pathway inactivation

Lijuan Liao,[†] Xiaolei Zhang,[†] Yi Lou, Chengzeng Zhou, Qianqian Yuan and Jiangtao Gao,[*]

College of Life Sciences, Fujian Agriculture and Forestry University, Fuzhou,350002 China

Table of Contents

page

Table S1. PCR primers used in this study 3

Figure S1. Diagnostic PCR strategy and results 4

Figure S2. The ^{1}H NMR (400 MHz, Methanol-d_4) spectrum of 8-methoxycichorine (**4**) 5

Figure S3. The ^{13}C NMR (100 MHz, Methanol-d_4) spectrum of 8-methoxycichorine (**4**) 5

Figure S4. The HSQC (400 MHz, Methanol-d_4) spectrum of 8-methoxycichorine (**4**) 6

Figure S5. The COSY (400 MHz, Methanol-d_4) spectrum of 8-methoxycichorine (**4**) 7

Figure S6. The HMBC (400 MHz, Methanol-d_4) spectrum of 8-methoxycichorine (**4**) 8

Figure S7. The UV spectrum of 8-methoxycichorine (**4**) 9

Figure S8. The HRESIMS spectrum in positive mode of 8-methoxycichorine (**4**) 9

Figure S9. The ^{1}H NMR (400 MHz, Methanol-d_4) spectrum of 8-*epi*-methoxycichorine (**5**) 10

Figure S10. The ^{13}C NMR (100 MHz, Methanol-d_4) spectrum of 8-*epi*-methoxycichorine (**5**) 10

Figure S11. The HSQC (400 MHz, Methanol-d_4) spectrum of 8-*epi*-methoxycichorine (**5**) 11

Figure S12. The COSY (400 MHz, Methanol-d_4) spectrum of 8-*epi*-methoxycichorine (**5**) 12

Figure S13. The HMBC (400 MHz, Methanol-d_4) spectrum of 8-*epi*-methoxycichorine (**5**) 13

Figure S14. The ^{1}H NMR (400 MHz, Methanol-d_4) spectrum of *N*-(4′-carboxybutyl)cichorine (**6**) 14

Figure S15. The ^{13}C NMR (100 MHz, Methanol-d_4) spectrum of *N*-(4′-carboxybutyl)cichorine (**6**) 14

Figure S16. The HSQC (400 MHz, Methanol-d_4) spectrum of *N*-(4′-carboxybutyl)cichorine (**6**) 15

Figure S17. The COSY (400 MHz, Methanol-d_4) spectrum of *N*-(4′-carboxybutyl)cichorine (**6**) 16

Figure S18. The HMBC (400 MHz, Methanol-d_4) spectrum of *N*-(4′-carboxybutyl)cichorine (**6**) 17

Figure S19. The UV spectrum of *N*-(4′-carboxybutyl)cichorine (**6**) 18

Figure S20. The HRESIMS spectrum in positive mode of *N*-(4′-carboxybutyl)cichorine (**6**) 18

Table S1 PCR primers used in this study.

Primers	Sequence(5'-3')	Application
An6448-L-AF	AGTCCCACCGTGCCTTTGTAGT	For deletion of Δ*An6448* gene
An6448-L-AR	ATAGCAACCATTTCGACGGAGATTCCAAAGGTCACTAAGTGTTGAAGG	
An6448-R-BF	ATCACGCATCAGTGCCTCCTCGAACTTCTTCCATCTCAGGTGTC	
An6448-R-BR	GGCTCGGATGATGATCTAACACT	
AfpyrG-CF	TCTCCGTCGAAATGGTTGCTAT	
AfpyrG-CR	GAGGAGGCACTGATGCGTGAT	
An6448-OF	ACGCCTGTGGGTGATCCTAT	Δ*An6448* mutant screen
An6448-OR	CCGCGTCATCTGGAGTGGTAAT	

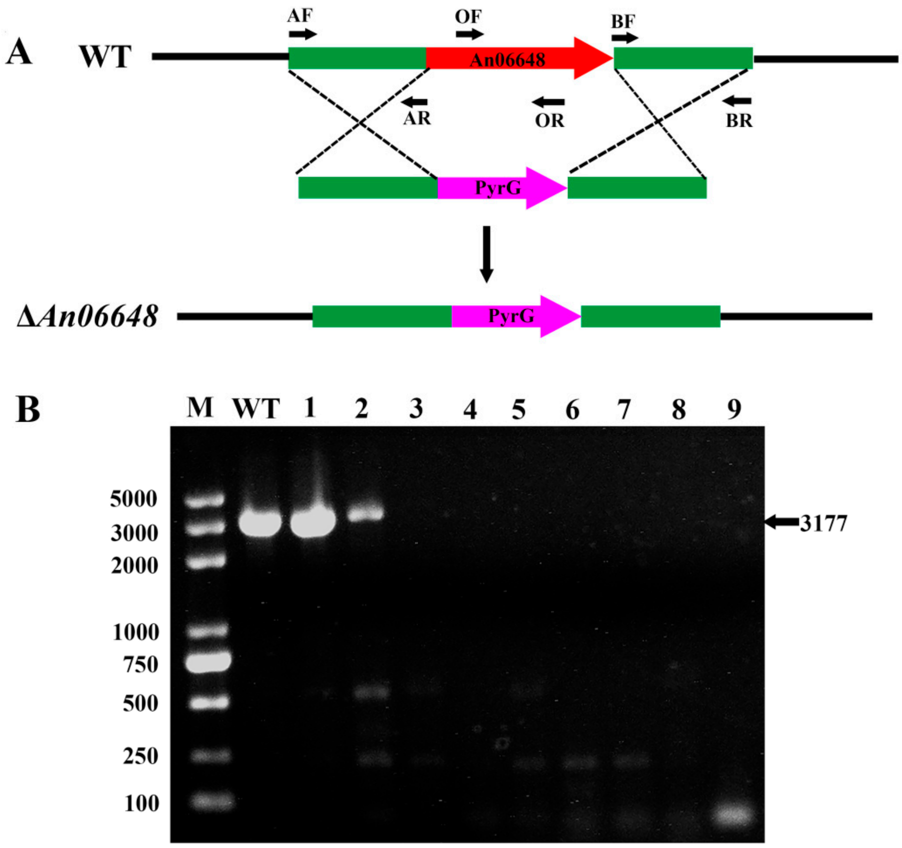

Figure S1. Gene knock-out of *An6448* in *A.nidulans* LO8030 (A) The *An6448*(*PkbA*) locus and gene replacement construct. The An6448 and PyrG are markered with red and magenta arrows, respectively. The primer pairs AF and AR, BF and BR were used to generated the gene replacement constructs. Primers OF and OR were used for mutant screening and identification. (B) Results of diagnostic PCR for the *AN6448* deletion Strains from number 3-9 were identified as the positive deletion mutants.

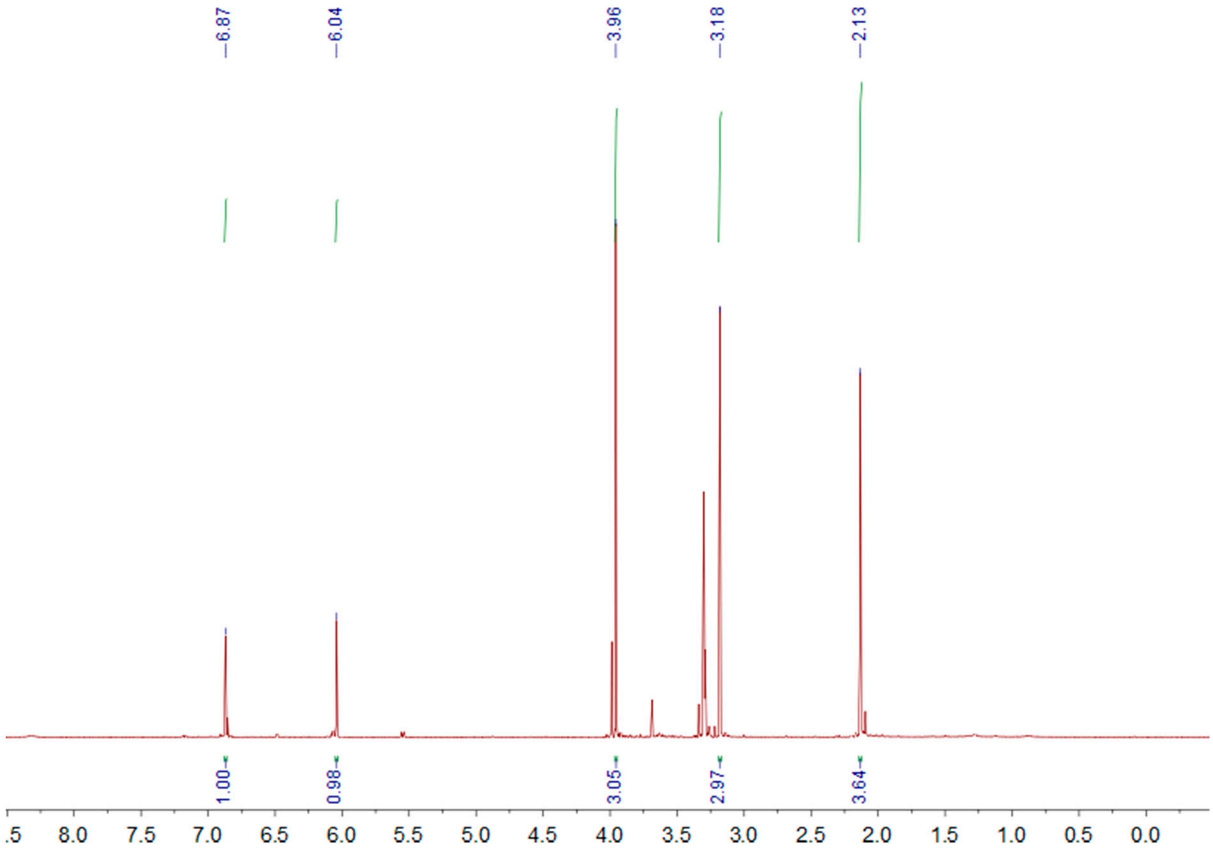

Figure S2. The ^{1}H NMR (400 MHz, Methanol-d_4) spectrum of 8-methoxycichorine (**4**)

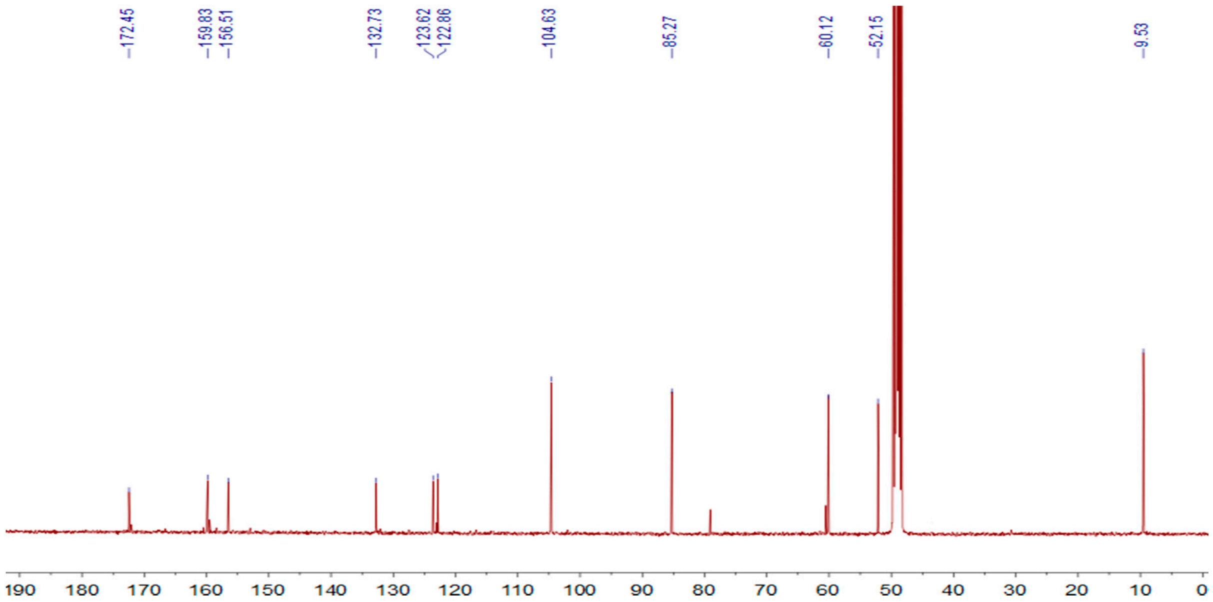

Figure S3. The ^{13}C NMR (100 MHz, Methanol-d_4) spectrum of 8-methoxycichorine (**4**)

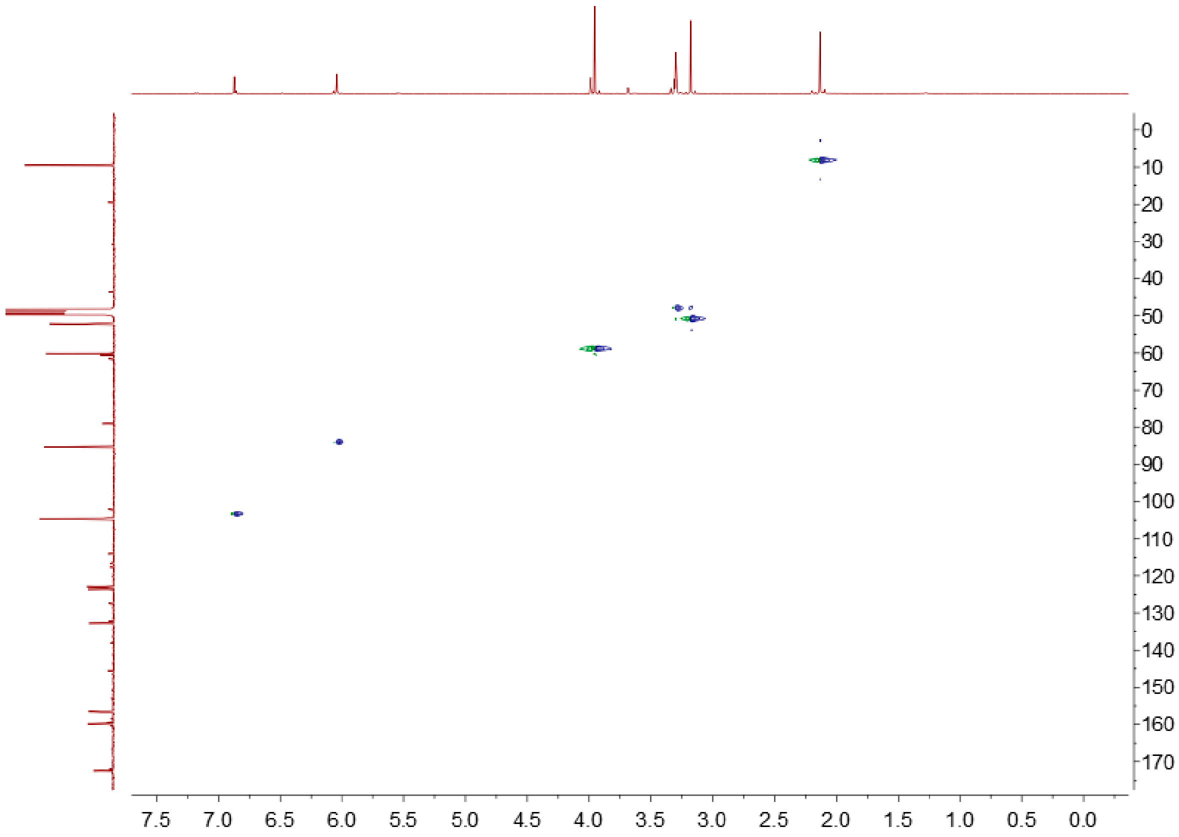

Figure S4. The HSQC (400 MHz, Methanol-d_4) spectrum of 8-methoxycichorine (**4**)

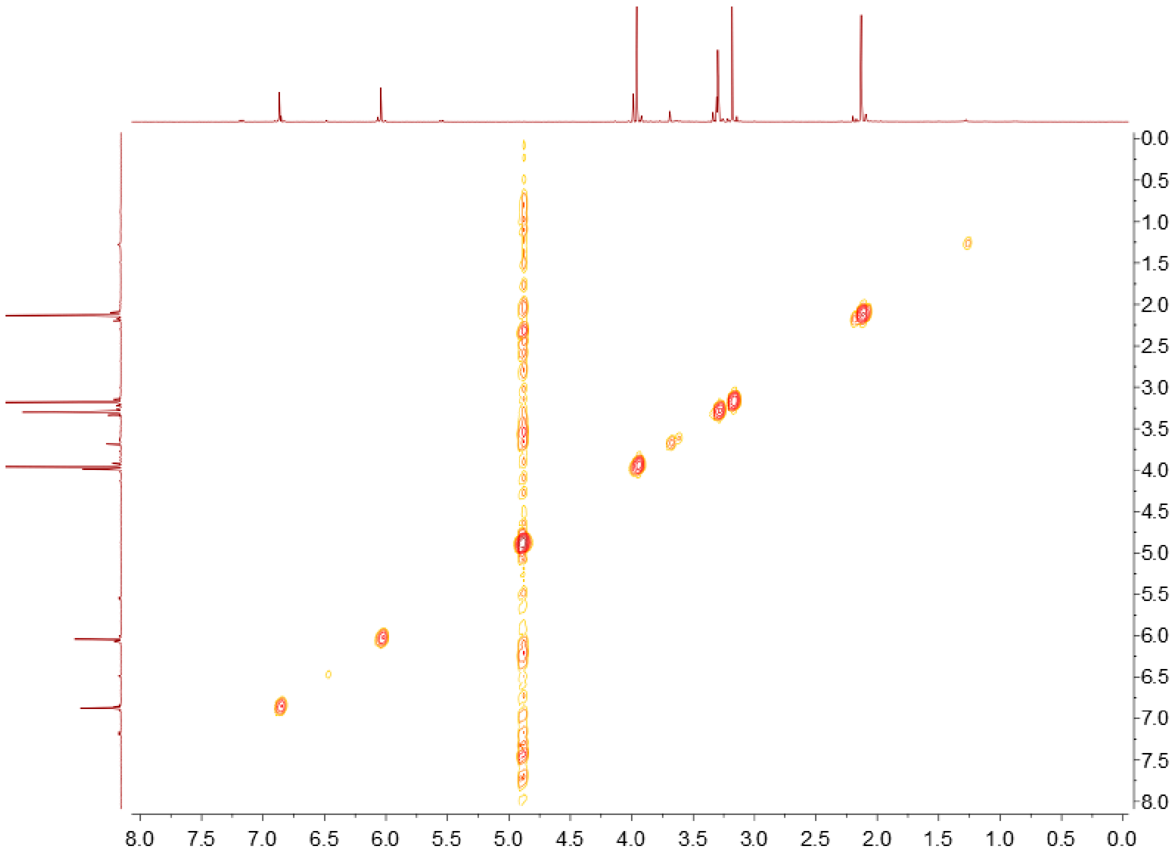

Figure S5. The COSY (400 MHz, Methanol-d_4) spectrum of 8-methoxycichorine (**4**)

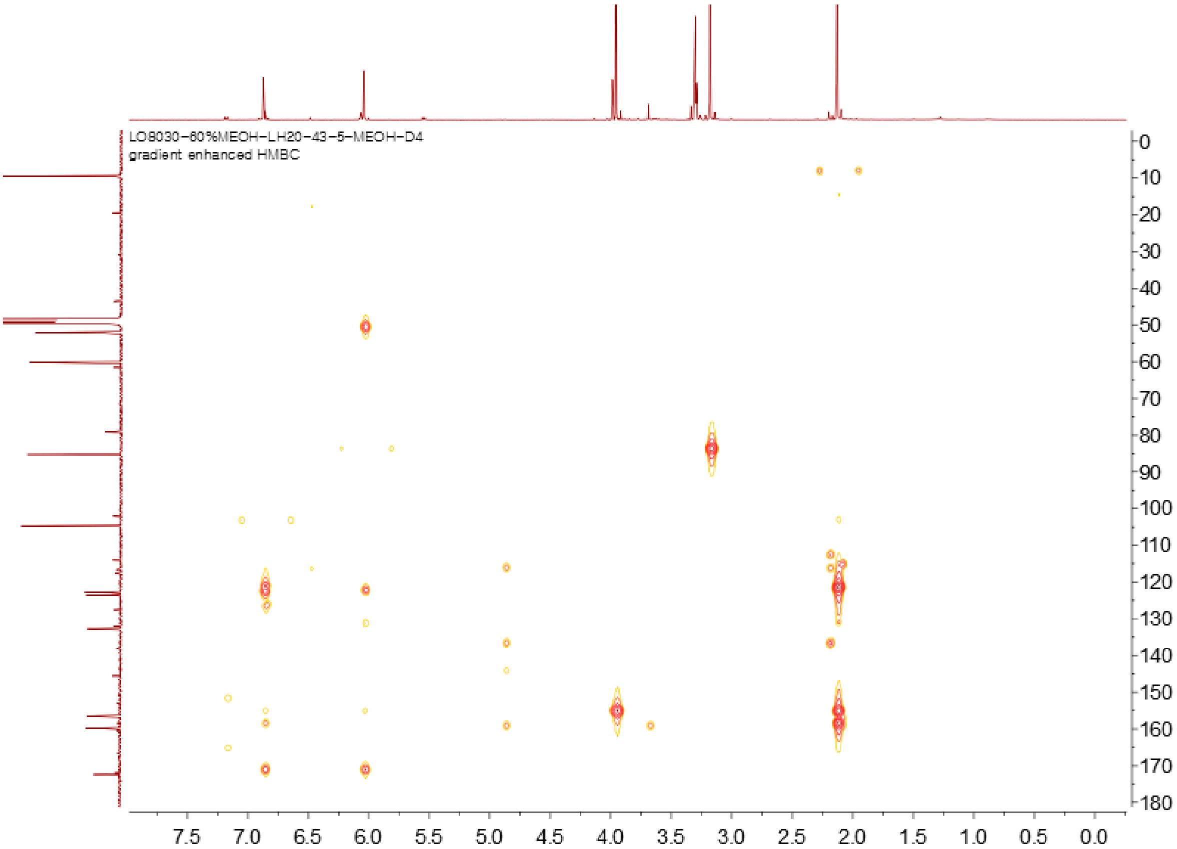

Figure S6. The HMBC (400 MHz, Methanol-d_4) spectrum of 8-methoxycichorine (**4**)

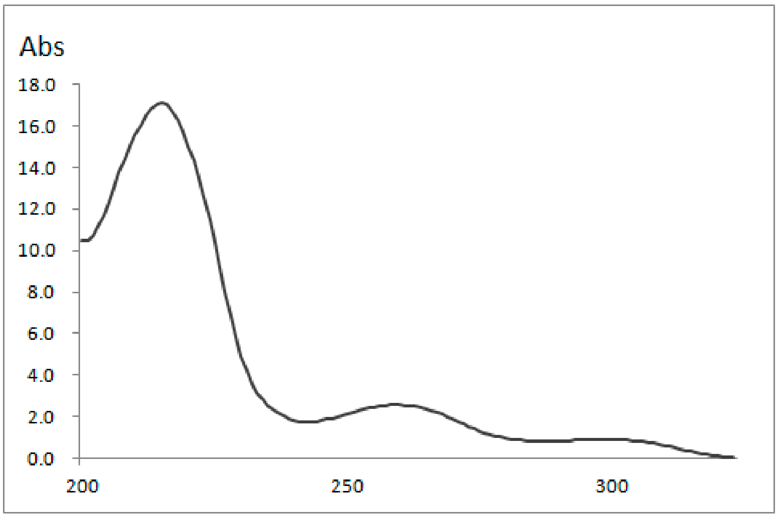

Figure S7. The UV spectrum of 8-methoxycichorine (**4**)

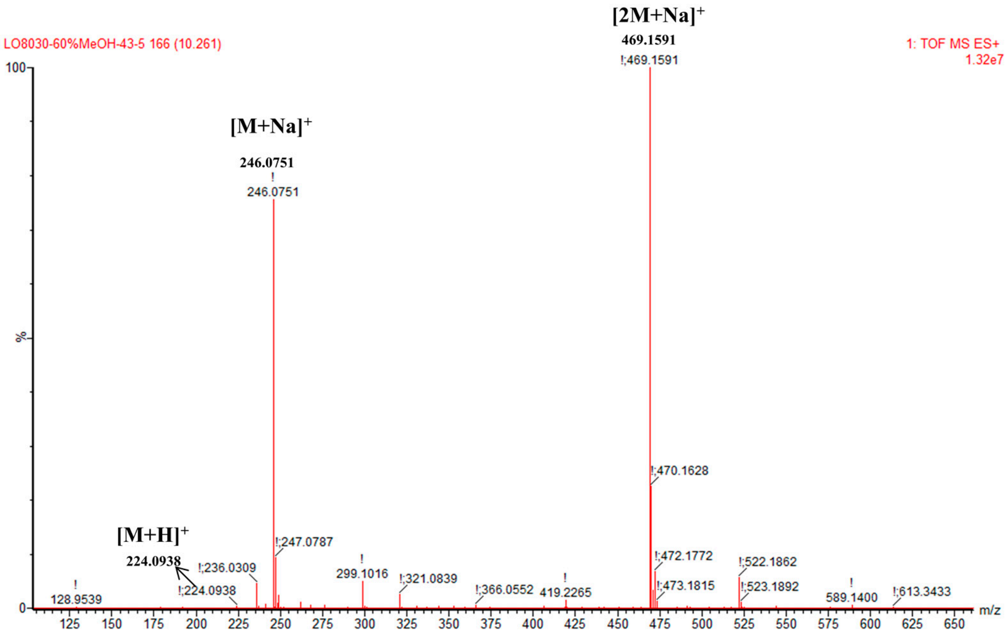

Figure S8. The HRESIMS spectrum in positive mode of 8-methoxycichorine (**4**)

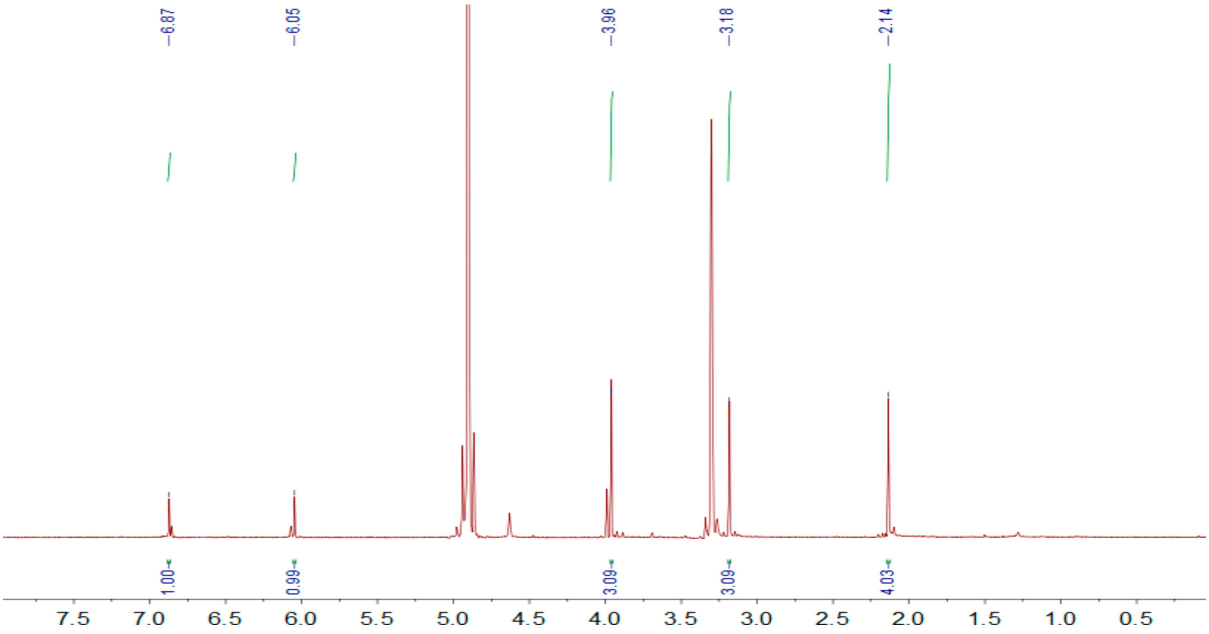

Figure S9. The ^{1}H NMR (400 MHz, Methanol-d_4) spectrum of 8-*epi*-cichorine (**5**)

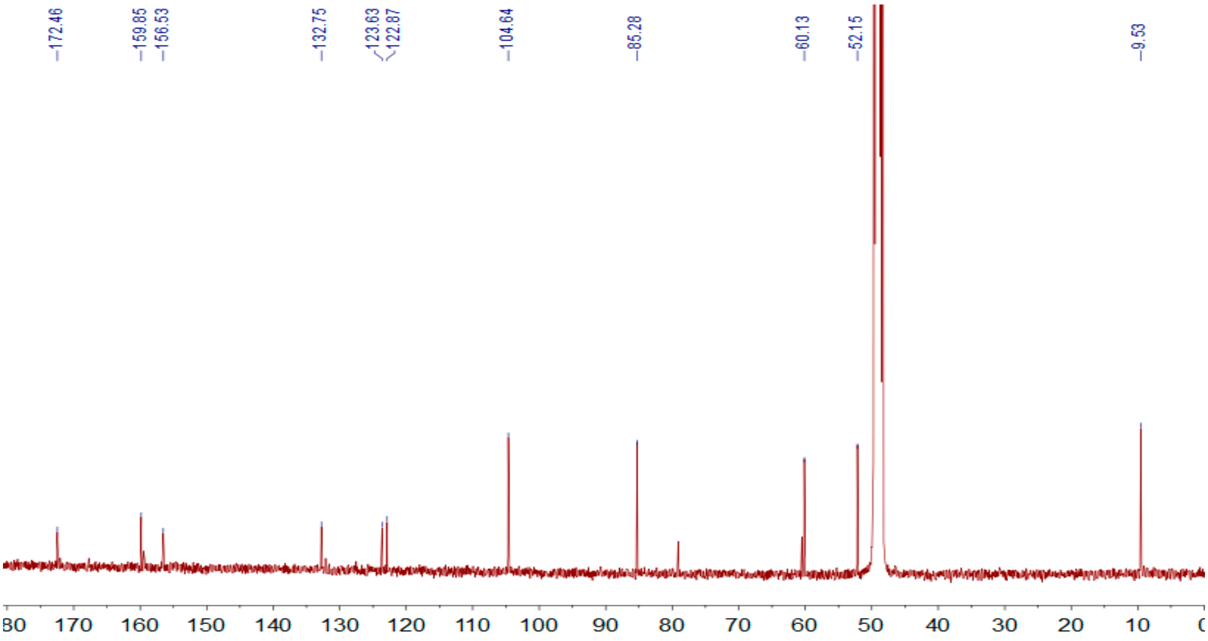

Figure S10. The ^{13}C NMR (100 MHz, Methanol-d_4) spectrum of 8-*epi*-cichorine (**5**)

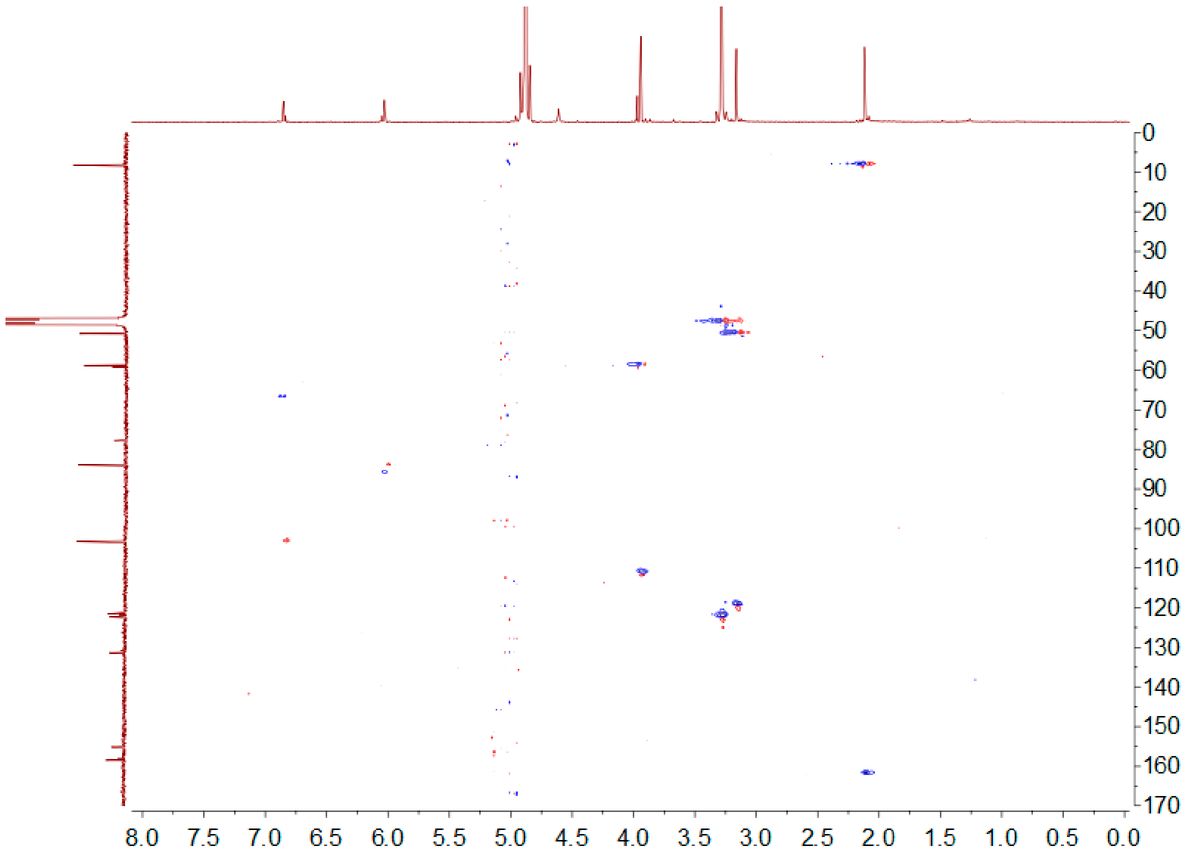

Figure S11. The HSQC (400 MHz, Methanol-d_4) spectrum of 8-*epi*-cichorine (**5**)

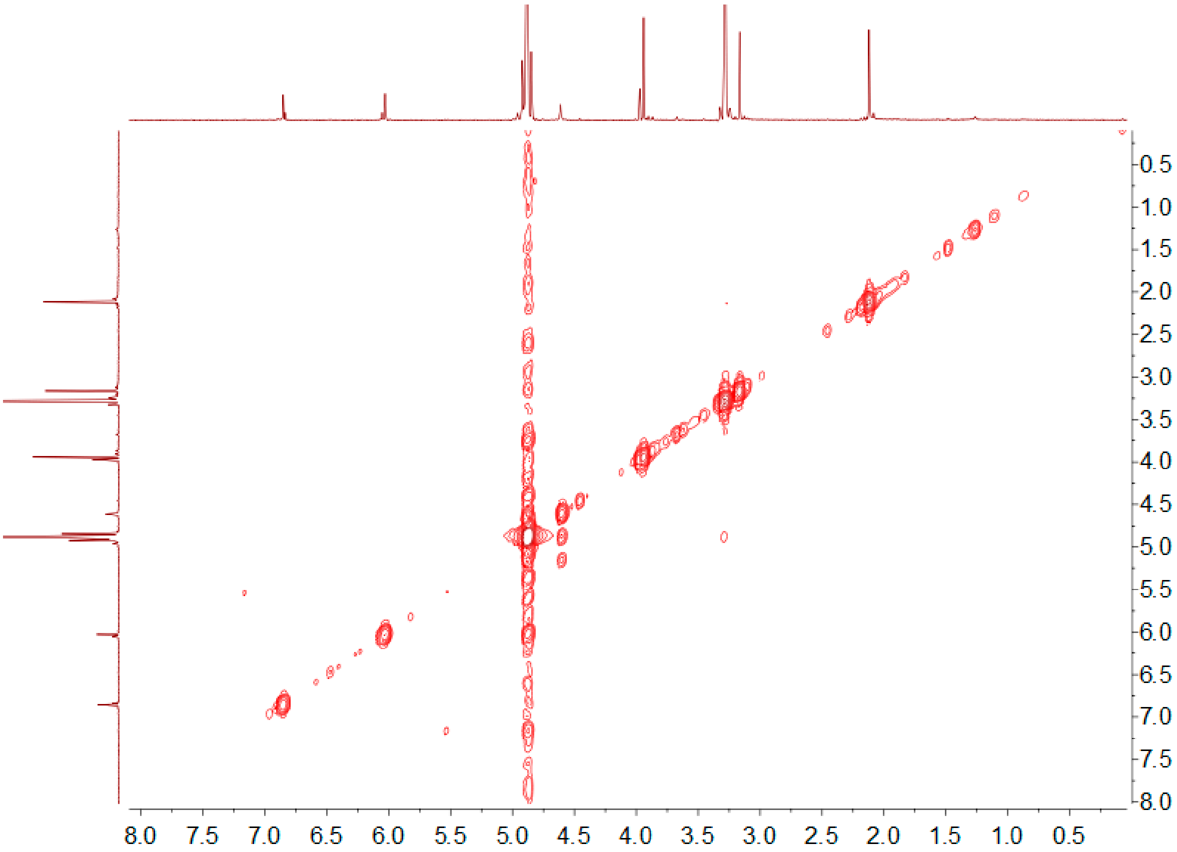

Figure S12. The COSY (400 MHz, Methanol-d_4) spectrum of 8-*epi*-cichorine (**5**)

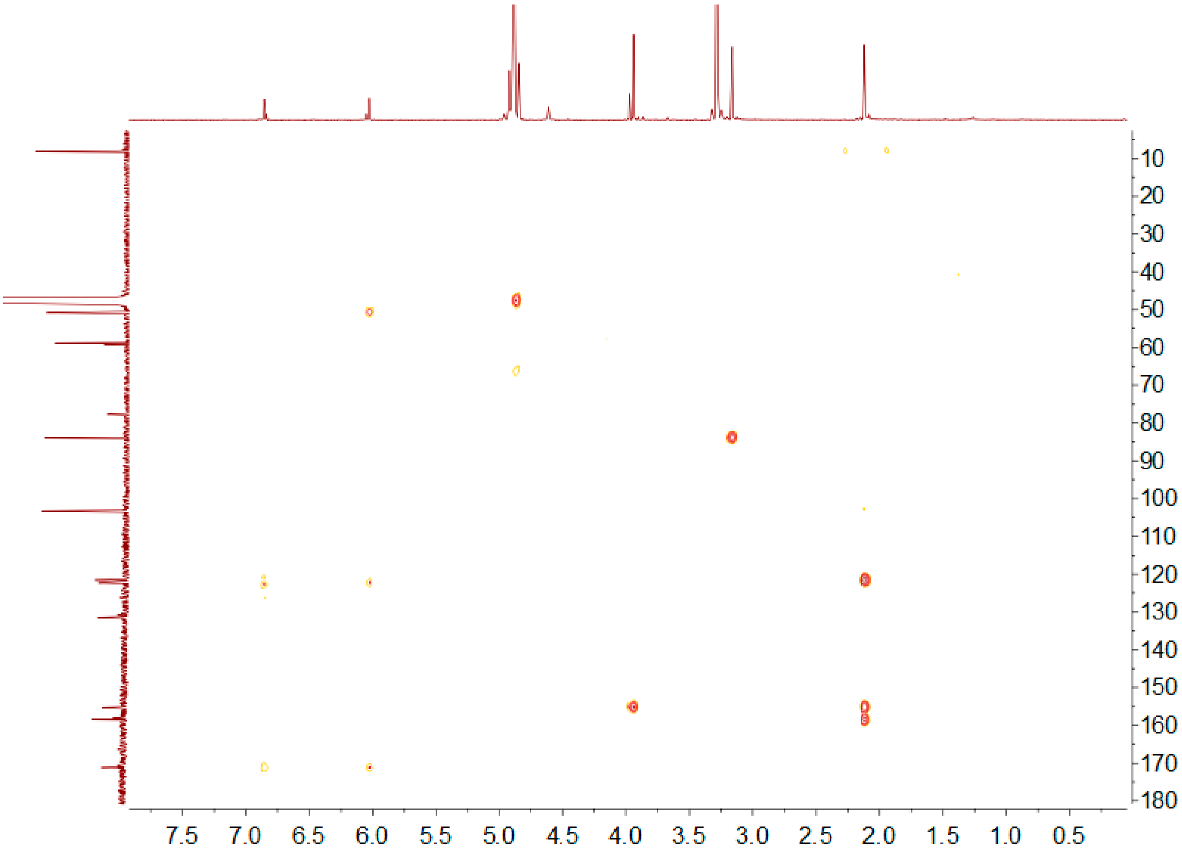

Figure S13. The HMBC (400 MHz, Methanol-d_4) spectrum of 8-*epi*-cichorine (**5**)

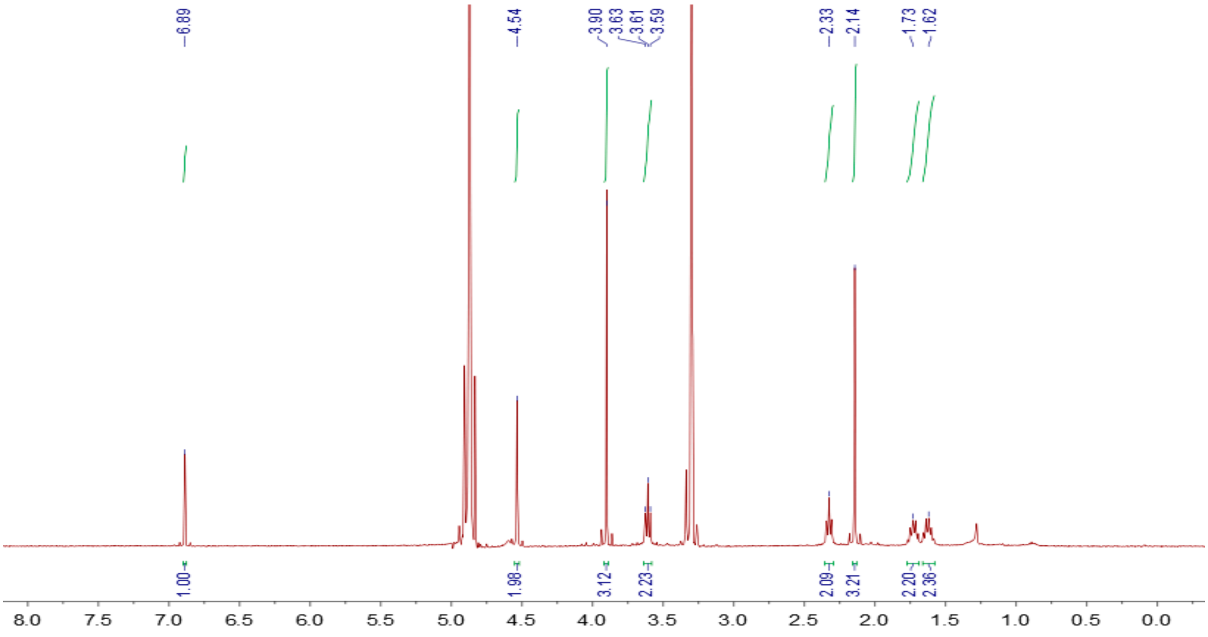

Figure S14. The ^{1}H NMR (400 MHz, Methanol-d_4) spectrum of *N*-(4′-carboxybutyl)cichorine (**6**)

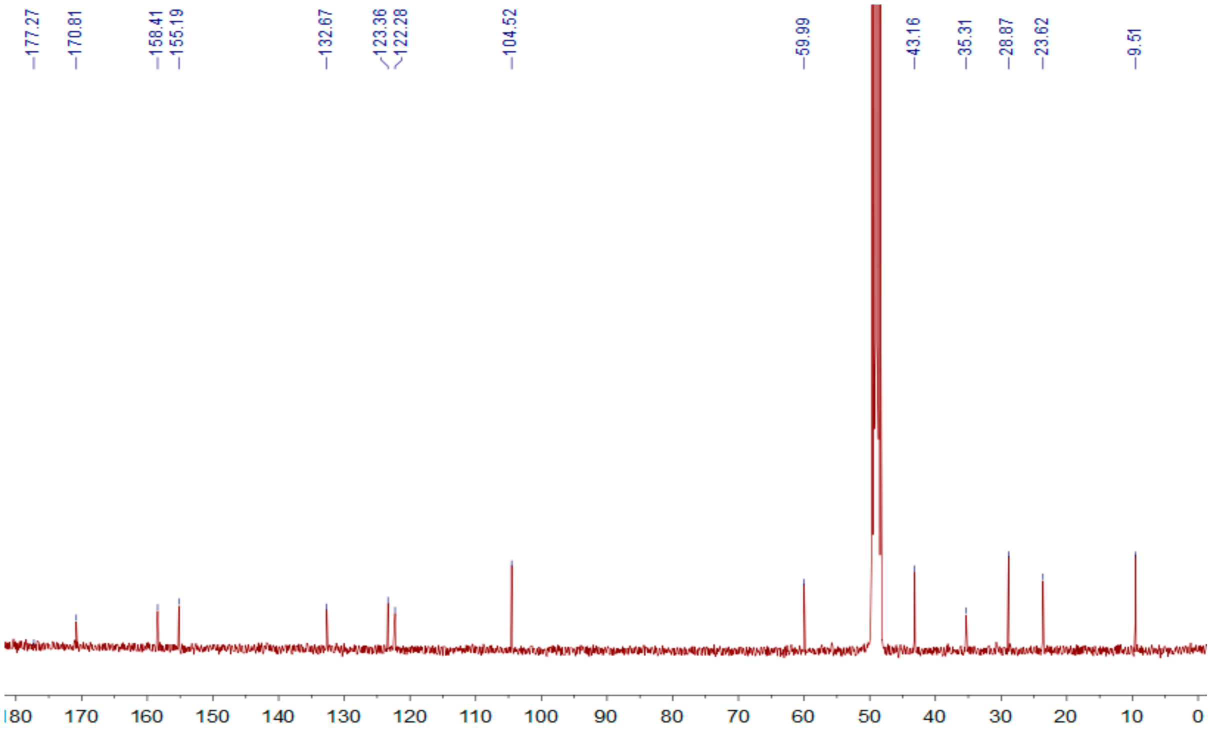

Figure S15. The ^{13}C NMR (100 MHz, Methanol-d_4) spectrum of *N*-(4′-carboxybutyl)cichorine (**6**)

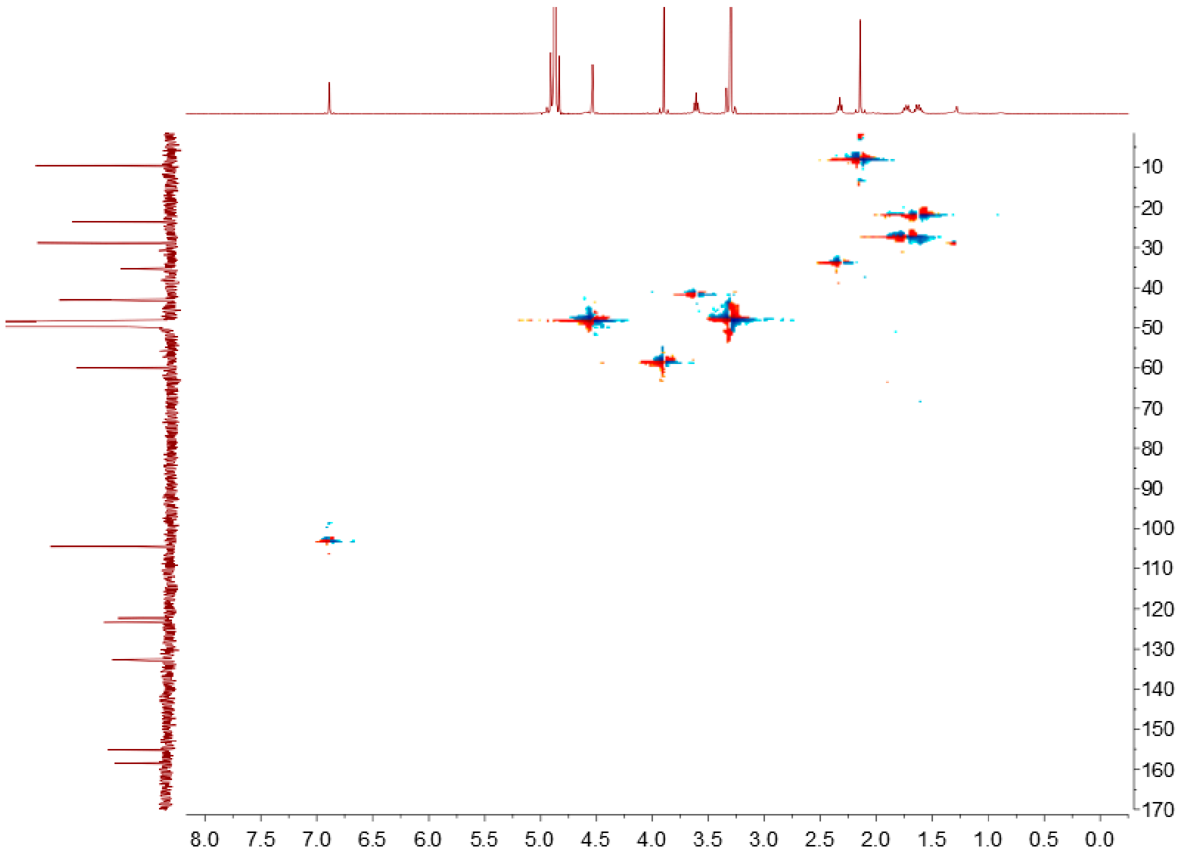

Figure S16. The HSQC (400 MHz, Methanol-d_4) spectrum of *N*-(4′-carboxybutyl)cichorine (**6**)

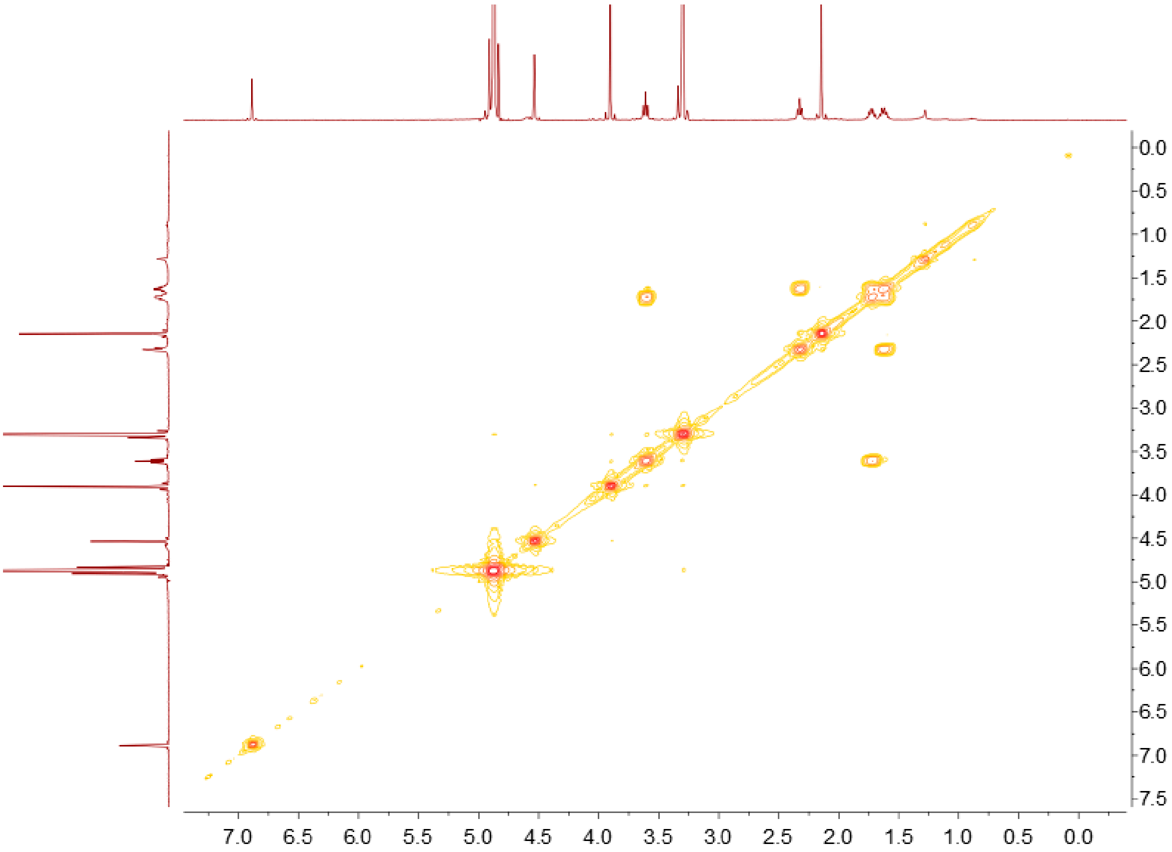

Figure S17. The COSY (400 MHz, Methanol-d_4) spectrum of *N*-(4′-carboxybutyl)cichorine (**6**)

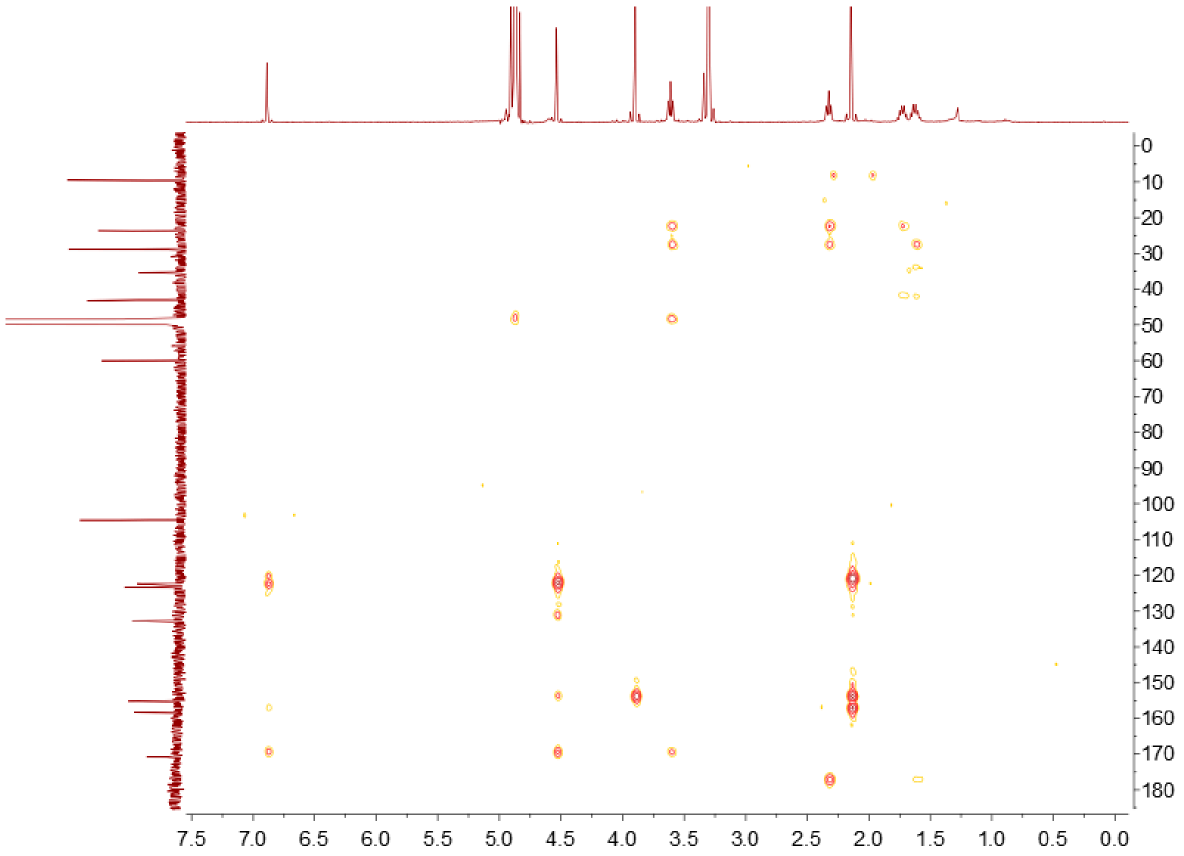

Figure S18. The HMBC (400 MHz, Methanol-d_4) spectrum of *N*-(4′-carboxybutyl)cichorine (**6**)

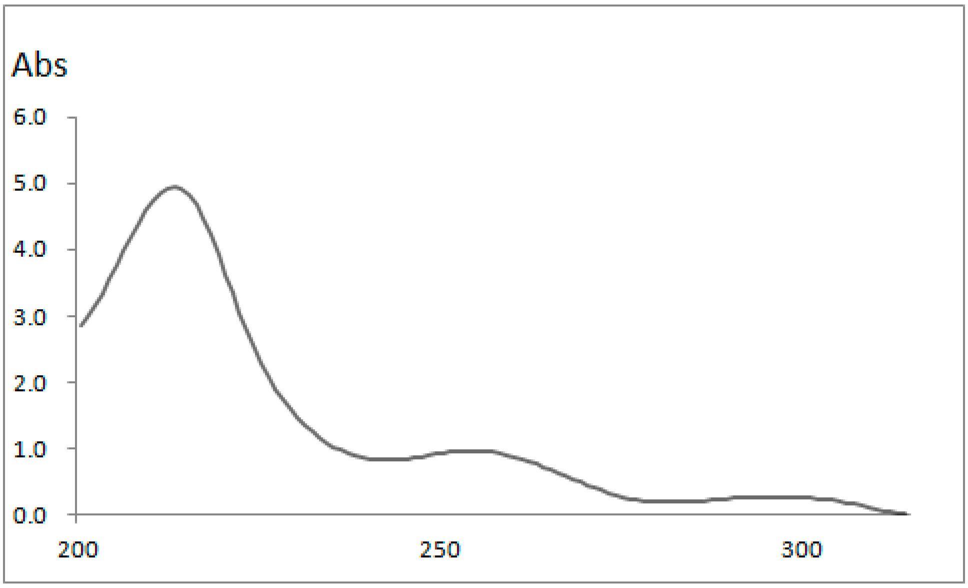

Figure S19. The UV spectrum of *N*-(4′-carboxybutyl)cichorine (**6**)

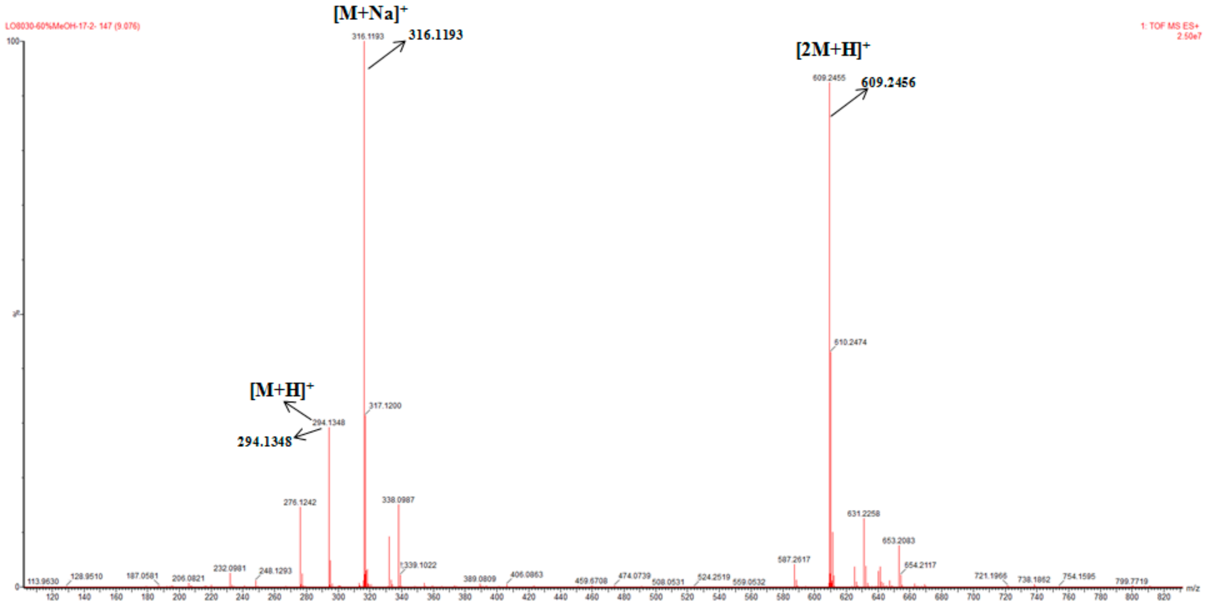

Figure S20. The HRESIMS spectrum in positive mode of *N*-(4′-carboxybutyl)cichorine (**6**)
